# Potential involvement of neutrophils in human thyroid cancer

**DOI:** 10.1371/journal.pone.0199740

**Published:** 2018-06-28

**Authors:** Maria Rosaria Galdiero, Gilda Varricchi, Stefania Loffredo, Claudio Bellevicine, Tiziana Lansione, Anne Lise Ferrara, Raffaella Iannone, Sarah di Somma, Francesco Borriello, Eduardo Clery, Maria Triassi, Giancarlo Troncone, Gianni Marone

**Affiliations:** 1 Department of Translational Medical Sciences (DiSMeT), University of Naples Federico II, Naples, Italy; 2 Center for Basic and Clinical Immunology Research (CISI), University of Naples Federico II, Naples, Italy; 3 WAO Center of Excellence, University of Naples Federico II, Naples, Italy; 4 Department of Public Health, University of Naples Federico II, Naples, Italy; 5 Department of Medicine, Division of Infectious Diseases, Boston Children’s Hospital and Harvard Medical School, Boston, Massachusetts, United States of America; 6 Institute of Experimental Endocrinology and Oncology “Gaetano Salvatore” (IEOS), National Research Council (CNR), Naples, Italy; Istituto Superiore di Sanità, ITALY

## Abstract

**Background:**

Neutrophil functions have long been regarded as limited to acute inflammation and the defense against microbes. The role(s) of neutrophils in cancer remain poorly understood. Neutrophils infiltrate tumors and are key effector cells in the orchestration of inflammatory responses. Thyroid cancer (TC) is the most recurrent endocrine malignant tumor and is responsible for 70% of deaths due to endocrine cancers. No studies are so far available on the role of neutrophils in TC.

**Objective:**

Our purpose was to study the involvement of tumor-associated neutrophils in TC.

**Methods:**

Highly purified human neutrophils (>99%) from healthy donors were stimulated *in vitro* with conditioned media derived from TC cell lines TPC1 and 8505c (TC-CMs). Neutrophil functions (e.g., chemotaxis, activation, plasticity, survival, gene expression, and protein release) were evaluated.

**Results:**

TC-derived soluble factors promoted neutrophil chemotaxis and survival. Neutrophil chemotaxis toward a TC-CM was mediated, at least in part, by CXCL8/IL-8, and survival was mediated by granulocyte-macrophage colony-stimulating factor (GM-CSF). In addition, each TC-CM induced morphological changes and activation of neutrophils (e.g., CD11b and CD66b upregulation and CD62L shedding) and modified neutrophils’ kinetic properties. Furthermore, each TC-CM induced production of reactive oxygen species, expression of proinflammatory and angiogenic mediators (CXCL8/IL-8, VEGF-A, and TNF-α), and a release of matrix metalloproteinase 9 (MMP-9). Moreover, in TC patients, tumor-associated neutrophils correlated with larger tumor size.

**Conclusions:**

TC cell lines produce soluble factors able to “educate” neutrophils toward an activated functional state. These data will advance the understanding of the molecular and cellular mechanisms of innate immunity in TC.

## Introduction

Thyroid cancer (TC) is a frequent solid tumor type worldwide and the most recurrent cancer of the endocrine system [1]. Indeed, TC is responsible for 90% of the endocrine malignant tumors and 70% of deaths due to endocrine tumors. In the past 5 years, the incidence of TC has progressively increased [2]. The prognosis of TC patients is highly variable, with small TCs showing only small possibility of tumor-specific morbidity or mortality, and with anaplastic TC being one of the most fatal solid tumors [3].

The relation between chronic inflammation and TC has long been described. Indeed, a combination of immune mediators and cellular effectors has been uncovered in TC and is related to tumor progression and clinical outcomes [4]. During activation of the MAPK and NF-κB pathways by oncogenic drivers, such as the RET/PTC rearrangement, RAS, and BRAF, thyrocytes are induced to produce a number of cytokines and chemokines that sustain tumor growth and progression [5,6,7]. Moreover, under resting conditions and/or as a consequence of proinflammatory stimuli, transformed thyrocytes produce and release inflammatory factors such as CXC chemokines (e.g., CXCL1, CXCL8, CXCL9, and CXCL10), which promote the recruitment and activation of tumor-infiltrating leukocytes [8,9,10,11].

Among tumor-infiltrating myeloid cells, macrophages are the best-characterized cells involved in tumor initiation and progression [12,13]. Tumor-associated macrophages (TAMs) manifest functional characteristics similar to those of alternative (M2) macrophages. In TC, TAMs show increased density and positively correlate with lymph node metastasis, larger tumor size, dedifferentiation, capsular invasion, extrathyroid extension, and reduced survival among the patients [14,15,16,17,18].

Neutrophils (polymorphonuclear leukocytes; PMNs) are well known leading actors in an acute inflammatory response and in the defense against extracellular microbes [19]. Nonetheless, a growing number of lines of evidence is shedding new light on the multiple roles of PMNs in the immune and inflammatory responses [12,20,21].

Indeed, studies have described the presence of tumor-associated neutrophils (TANs) in cancer, which correlate with patients’ clinical outcomes [22,23,24,25,26,27,28]. Nevertheless, their functional roles at the various steps of tumor initiation and progression are still a matter of debate. For instance, TANs have been associated with genetic instability and neutrophil-derived cytokines (e.g., OSM, VEGF) or granule proteins (e.g., neutrophil elastase) play many roles in the promotion of cancer cell proliferation, invasive behavior, and the angiogenic switch [29,30,31,32,33]. In contrast, antitumor neutrophils were recently proposed that can kill tumor cells, to stimulate the T- cell–dependent anti-tumoral immunity, and inhibit angiogenesis have been recently proposed [28,34,35,36]. Therefore, to date, the participation of neutrophils in different types of cancer is still controversial and its deciphering remains an important challenge.

The prognosis of patients with TC remains difficult because of heterogeneity of this pathology manifesting distinct clinical and molecular characteristics [37]. The ratio of the peripheral-blood neutrophil count to the lymphocyte count (neutrophil-to-lymphocyte ratio; NLR) has been associated with tumor development and progression [38]. In patients with TC, a higher NLR correlates with larger tumor volume and higher risk of recurrence but is not effective at discriminating benign from malignant lesions [39]. Moreover, the NLR does not correlate with the risk of occult metastasis or with patients’ survival [40]. Thus, the prognostic significance of NLR in TC remains uncertain. To our knowledge, there is no information concerning the occurrence, functions, and prognostic significance of TANs in TC.

In this study, for the first time, we investigated the presence of infiltrating neutrophils in human TC and analyzed the phenotypic and functional characteristics of “tumor-educated” neutrophils. In particular, we took advantage of an *in vitro* model to elucidate the functional interactions between TC cells and human neutrophils. We found that TC cells recruited neutrophils and significantly improved their survival. Moreover, TC cells upregulated neutrophils’ proinflammatory activities as well as the expression of factors that can retain the ability to promote tumor progression. Finally, we found that PMNs infiltrated human TC and correlated with tumor size, further supporting the potential tumor-promoting role of TANs in TC.

## Materials and methods

### Cell cultures and preparation of tumor-conditioned media

Human thyroid tumor cell lines TPC1 (papillary thyroid cancer), 8505c (anaplastic thyroid cancer) and Nthy-ori 3–1 (immortalized thyroid follicular epithelial cell line derived from normal adult thyroid tissue that has been transfected with a plasmid encoding the SV40 large T gene) were from ATCC, cultured and maintained in DMEM supplemented with 10% of heat-inactivated fetal calf serum (FCS; endotoxin level <0.1 EU/ml), 50 U/ml penicillin/streptomycin, and 2 mM L-glutamine (Euroclone, Milan, Italy) at 37°C in a humidified atmosphere containing 5% of CO_2_ and 95% of air. Conditioned media were prepared and used as follows. Cells were seeded at 10–20% confluence in tissue culture plates. Once the cells reached confluence of 85–90%, the cell culture medium was replaced with a serum-free fresh medium. After 24 hours, this conditioned medium was harvested, filtered (0.20 μm pore size filter), and stored at −20°C. All the cell lines were routinely checked for mycoplasma contamination.

### Neutrophil purification and culture

The study protocol involving the use of human blood cells was approved by the Ethical Committee of the University of Naples Federico II, and written informed consent was obtained from blood donors according to the principles expressed in the Declaration of Helsinki. Granulocytes were isolated from buffy coats of healthy donors (HBsAg^−^, HCV^−^, and HIV^−^) obtained from a leukapheresis unit. Leukocytes were separated from erythrocytes by dextran sedimentation. Neutrophils were purified by Ficoll-Paque Histopaque^®^-1077 (Sigma Aldrich, Milan, Italy) density gradient centrifugation (400 × *g* for 30 minutes at 22°C), followed by Percoll (Sigma Aldrich, Milan, Italy) (65%) density gradient centrifugation (660 × *g* for 20 minutes at 22°C), as previously described [41]. Finally, neutrophils were isolated from granulocytes (to reach >99% purity) by positive elimination of all contaminating cells using the EasySep Neutrophil Enrichment Kit (StemCell Technologies, Vancouver, Canada) [42].

These cells were >99% neutrophils as evaluated by flow-cytometric analysis with the following antibodies: anti-CD3, anti-CD14, anti-CD15, anti-CD11b, anti-CD193 (Miltenyi Biotec, Germany), anti-CD62L (L-Selectin) (BD Biosciences, USA), and anti-CD66b (Biolegend, CA, USA). Samples were analyzed on the MACSQuant Analyzer 10 (Miltenyi Biotec, Germany) and in the FlowJo software, v.10. Doublets and debris were excluded from the analysis. Data were expressed as a percentage of positive cells or median fluorescence intensity [43]. Spontaneous activation of neutrophils was evaluated by analyzing CD11b and L-selectin expression by flow-cytometric analysis before and after neutrophil purification; only L-selectin^+^CD11b^low^ (nonactivated) neutrophils were chosen for the study (data not shown).

### Quantification of soluble factors in culture supernatants or total protein lysates

CXCL8/IL-8, granulocyte-macrophage colony-stimulating factor (GM-CSF), and MMP-9 concentrations in cell-free conditioned media or total protein lysates (0.1% Triton X-100) were assessed in duplicate with commercially available ELISA kits (R&D Systems). MMP-9 levels in total protein lysates were normalized to total protein concentrations as determined by a Bradford protein assay (Bio-Rad) and expressed in nanograms of protein per milligram of total protein. A microplate reader (Tecan, Austria, GmbH) was used to determine sample absorbance at 450 nm. The ELISA sensitivity is 31.1–2.000 pg/ml for CXCL8/IL-8, 15.6–1000 pg/ml for GM-CSF, and 31.3–2000 pg/ml for MMP-9.

### Cell migration assay

Migration of neutrophils toward TC-conditioned media (TC-CMs) was evaluated by means of a 3 μm cell culture inserts in 96-well companion plates (Corning Costar). The companion plates were loaded with 235 μl of a conditioned medium or control medium (serum-free DMEM). PMNs (2.5 × 10^6^ neutrophils/ml per 75 μl) were placed in the inserts and allowed to migrate at 37°C and 5% CO_2_ for 1 hour. At the end of the incubation, the cells were centrifuged and resuspended in 100 μl of PBS and counted by flow cytometry (MACSQuant Analyzer 10, Miltenyi Biotec, Germany). In some experiments, neutrophils were preincubated with mouse monoclonal anti-CXCR1 and/or anti-CXCR2 blocking antibodies at 10 μg/ml (clone 42705 and clone 48311 respectively, R&D Systems) or the corresponding control isotype (R&D Systems) at 37°C and 5% CO_2_ for 60 minutes and then subjected to the migration assay as already described above.

### Apoptosis assay and morphological analysis of neutrophils

Purified neutrophils (2 × 10^6^ cells/ml) were cultured in a TPC1 or 8505c conditioned medium with or without the mouse monoclonal anti-GM-CSF blocking antibody at 10 μg/ml (clone 3209, R&D Systems) or the corresponding control isotype (R&D Systems). For each time point, neutrophils were stained with fluorescein isothiocyanate (FITC)-conjugated annexin V and propidium iodide (PI) according to the protocol provided by the manufacturer (Miltenyi Biotec, Germany). Quantification was performed on a MACS Quant flow cytometer (Miltenyi Biotec, Germany). Live cells were assumed to be double-negative annexin V^−^PI^−^ cells. Analysis was performed by means of FlowJo v.10.

### Flow cytometry

These experiments were conducted with purified neutrophils. For activation experiments, the cells were kept in RPMI 1640 with 10% of FCS for 1 h and stimulated with one of TC-CMs for 90 minutes. Then, the cells were stained (20 minutes, 4°C) in PBS plus 1% FCS (Euroclone, Milan, Italy) (staining buffer containing antibodies). The following antibodies were employed: VioBlue-conjugated anti-human CD15 (clone VIMC6, dilution 1:10, from Miltenyi Biotech, Germany), phycoerithrin (PE)-conjugated anti-human CCR3 (clone 5E8, dilution 1:10, from Biolegend, CA, USA), allophycocyanin (APC)-conjugated anti-human CD66b (clone G10F5, dilution 1:20, from Biolegend, CA, USA), APC-conjugated anti-human CD11b (clone ICRF44, dilution 1:50, from eBiosciences), and FITC-conjugated anti-human CD62L (clone DREG-56, dilution 1:10, from BD Biosciences, USA). The samples were studied on the MACS Quant Analyzer 10 (Miltenyi Biotec) and in FlowJo v.10. Doublets and debris (identified based on forward and side scatter properties) were excluded from the analysis. Data are expressed as a percentage of positive cells or median fluorescence intensity [43].

### Reactive oxygen species (ROS) production

Neutrophils (2 × 10^6^ cells/ml) were resuspended in the RPMI 1640 medium with 2% of fetal bovine serum (FBS) and antibiotics at 37°C and 5% CO_2_. The cells were incubated for 30 minutes after the addition of 10 μg/ml H_2_DCF-DA (Life Technologies, Milan, Italy). H_2_DCF-DA is a fluorogenic dye that allows researchers to determine hydroxyl peroxyl and other ROS activities within the cell. Once diffused into the cell, H_2_DCF-DA is deacetylated by cellular esterases to a nonfluorescent molecule, which is oxidized by ROS into 2′,7′dichlorofluorescein (DCF). DCF is highly fluorescent and can be detected by fluorescence spectroscopy with maximum excitation and emission wavelengths of 492–495 and 517–527 nm, respectively. The cells were washed in PBS and resuspended in a TC-CM, control medium, or phorbol myristate acetate (PMA; 10 ng/ml) and immediately seeded in a 96-well plate and placed in a EnSpire Multimode Plate Reader (Perkin Elmer). DCF mean fluorescence intensity was measured at an excitation wavelength of 492–495 nm and emission at 517–527 nm. The ability of a TC-CM to induce cytoplasmic ROS-catalyzed oxidation of DCFH in neutrophils was measured as compared to the positive control (PMA; Sigma-Aldrich, Milan, Italy) and to the negative control (the medium alone).

### RNA isolation and real-time RT-PCR

Total RNA was extracted with the TRIzol Reagent (Thermo Fisher Scientific) and quantified on a Nanodrop ND-1000 spectrophotometer (Thermo Scientific, Wilmington, DE, USA). Reverse transcription was performed using the High-Capacity cDNA Reverse Transcription Kit (Applied Biosystems, Foster City CA, USA). Real-time RT-PCR was performed by means of Universal SYBR Green Supermix (Bio-Rad) on a CFX96 Real-time detection system (Bio-Rad). Relative gene expression was calculated by the ΔC_t_ (relative expression) method. Each C_t_ value was normalized to the respective *GAPDH* C_t_ value. Target-specific primers for *GAPDH*, *CXCL8/IL-8*, *TNF-α*, and *VEGF-A* were synthesized and purified by Custom Primers (Life Technologies, Milan, Italy).

### Fluorescence, time-lapse, and high-content microscopy

Microscopy experiments were conducted with the Operetta High-Content Imaging System (PerkinElmer), similarly to previously described procedures [43]. Neutrophils were cultured in 96-well black CellCarrier plates (PerkinElmer). For time-lapse experiments, neutrophils were cultured overnight. Within this time window, digital phase contrast images of 15 fields/well were captured every 15 minutes via a 20× objective. To quantify cell morphological features, bright-field snapshots were taken at 15 fields/well. PhenoLOGIC (PerkinElmer) was employed for image segmentation and for calculating the single-cell morphological results by the dedicated STAR analysis sequence [43]. STAR morphology is an enhanced series of algorithms that provide a statistically powerful set of properties for analyzing phenotypes by characterizing cell morphology and the distribution of intensity within regions. The STAR method offers the possibility to calculate symmetry properties, threshold compactness, axial properties, radial properties, and a profile [43,44].

### Immunohistochemistry

We retrieved 32 thyroid tumors from the archives of the Department of Public Health, Pathology Division, University of Naples Federico II. These cases included papillary thyroid carcinomas (n = 23), follicular adenomas (n = 1), Hürthle cell adenomas (n = 4), follicular carcinoma (n = 3), and one case of medullary thyroid carcinoma. Four-micron slices of formalin-fixed paraffin-embedded cell blocks were placed on charged slides, then deparaffinized and dehydrated. To detect the infiltrating neutrophils, we employed the anti-CD66b monoclonal antibody (clone G10F5, dilution 1:100) as a primary antibody [22,27,28]. After heat-induced antigen retrieval, the slides were processed by Benchmark XT Autostainer (Ventana, Roche) using the UltraView Polymer Detection kit. Negative controls were implemented by omitting the primary antibody. Whole-tumor section CD66b^+^ neutrophils were counted and scored by a trained pathologist at 200× magnification. Only CD66b^+^ neutrophils infiltrating the tumorous lesions were considered, avoiding those within the vascular spaces. The CD66b^+^ neutrophil count in tumor samples was distributed according to the tumor size. The median value of tumor size served as a cutoff. The numbers of CD66b^+^ neutrophils were also studied regarding a possible correlation with the dimensions (in cm) of each thyroid nodule.

### Statistical analysis

The data are expressed as mean ± SEM of the indicated number of experiments. Statistical analysis was performed in Prism 6 (GraphPad Software). Values from groups were compared by Student’s *t* test or repeated-measures one-way or two-way analysis of variance corrected for multiple comparisons as appropriate. Pearson’s analysis was carried out to test the correlation between CD66b^+^ tumor infiltrating neutrophils and tumor size. Differences were assumed to be statistically significant when the *p* value was < 0.05.

## Results

### TC-derived soluble mediators induced neutrophil chemotaxis

**Fig 1:**
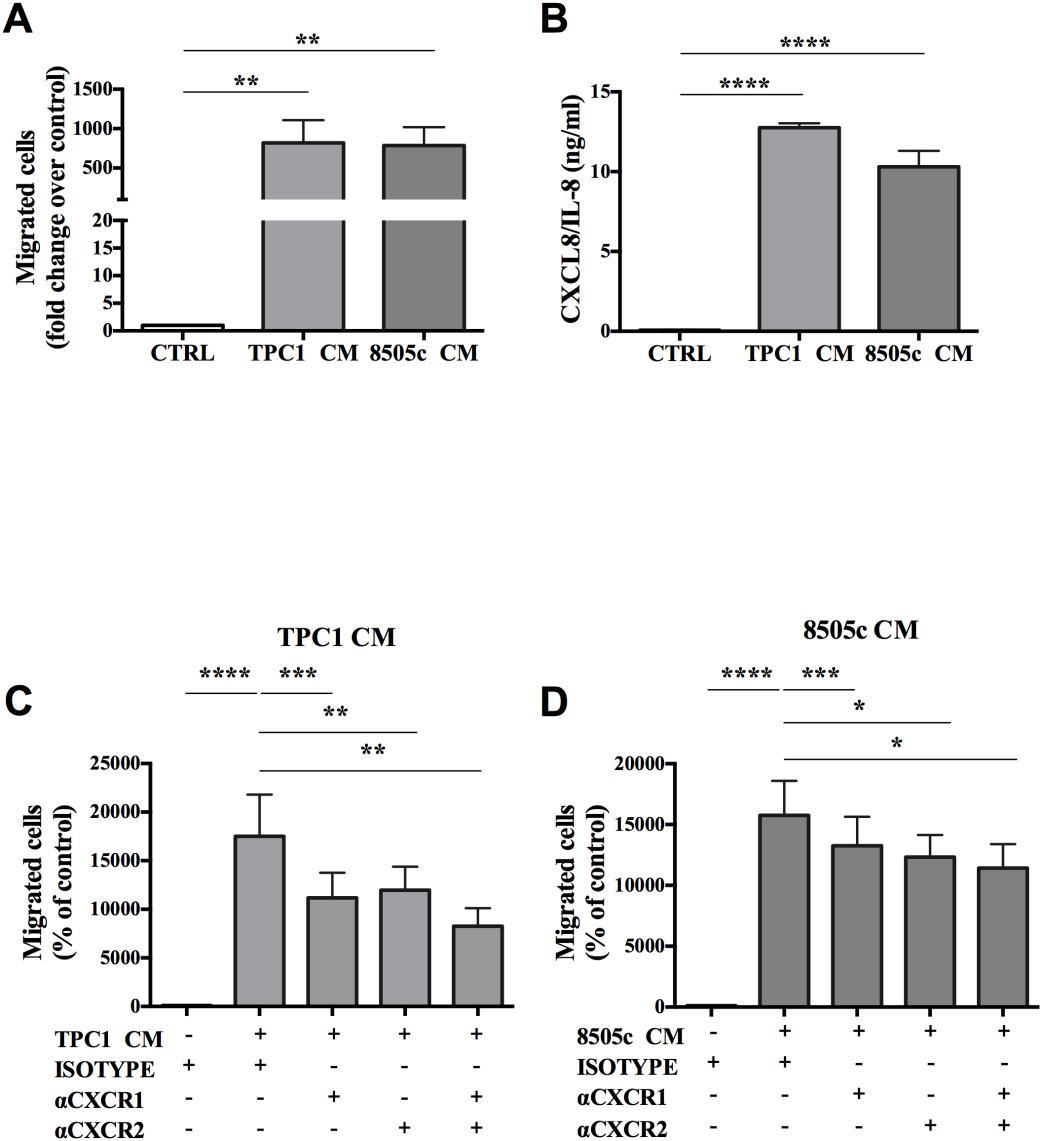
TC-derived soluble mediators induced neutrophil chemotaxis. **A.** Neutrophil chemotaxis toward TC-CM or the control medium was evaluated using 3 μm cell culture inserts in 96-well companion plates. Neutrophils (2.5 × 10^6^ cells/ml per 75 μl) were allowed to migrate (37°C, 60 minutes) toward a TC-CM or the control medium (235 μl per well). At the end of the incubation, the cells were centrifuged and resuspended in PBS (100 μl) and counted by flow cytometry. Data are expressed as migratory cells relative to the control (mean ± SEM of five independent experiments), **p < 0.01. **B.** The CXCL8/IL-8 release by TPC1 and 8505c cells was evaluated by an ELISA in a TC-CM or in the control medium. Results are expressed as mean ± SEM of seven independent experiments; ****p < 0.001. **C and D**. Chemotactic activity of neutrophils *via* a TPC1-derived **(C)** or 8505c-derived **(D)** conditioned medium was analyzed in the presence of blocking antibodies directed against CXCR1 and/or CXCR2 (10 μg/ml) or the related isotype control. Migratory neutrophils were counted by flow cytometry. The results are expressed as a percentage of isotype control (mean ± SEM of eight independent experiments); ***p < 0.005; **p < 0.01; *p < 0.05.

In the first group of *in vitro* experiments, we studied the ability of TC cell lines to direct migration of PMNs, referred to as chemotaxis. Highly purified human PMNs from peripheral blood of healthy donors were allowed to migrate toward a TC-CM from papillary TC cell line TPC1 or from the anaplastic TC cell line 8505c or toward a control medium. After 1 hour of incubation, migrating cells were counted by flow cytometry. TC-CM was found to induce greater directed migration of PMNs as compared to the control medium (Fig 1A). These results suggested that soluble factors released by TC induced PMN chemotaxis.

Thyroid cancer cell lines autocrinously produce a large amount of CXCL chemokines [8,10,45], which can be responsible for neutrophil chemotaxis [20,46]. CXCL8/IL-8 was found in large amounts (~10 ng/ml) in TC-CM (Fig 1B). CXCL8/IL-8 retains a well-known chemotactic activity for neutrophils, acting through CXCR1/2 and playing a pivotal role in the tumor microenvironment (TME) [47,48]. To investigate the mechanisms of PMN chemotaxis, neutralizing antibodies against CXCL8/IL-8 receptors CXCR1 and/or CXCR2 were used. Thus, PMNs were allowed to migrate toward a TC-CM in the presence of a CXCR1-blocking and/or CXCR2-blocking antibody or the related isotype (control). The results showed that blocking of CXCR1 and CXCR2 significantly reduced PMN chemotaxis toward a TC-CM **(**Fig 1C and 1D).

### TC-derived soluble factors promoted neutrophil survival

**Fig 2:**
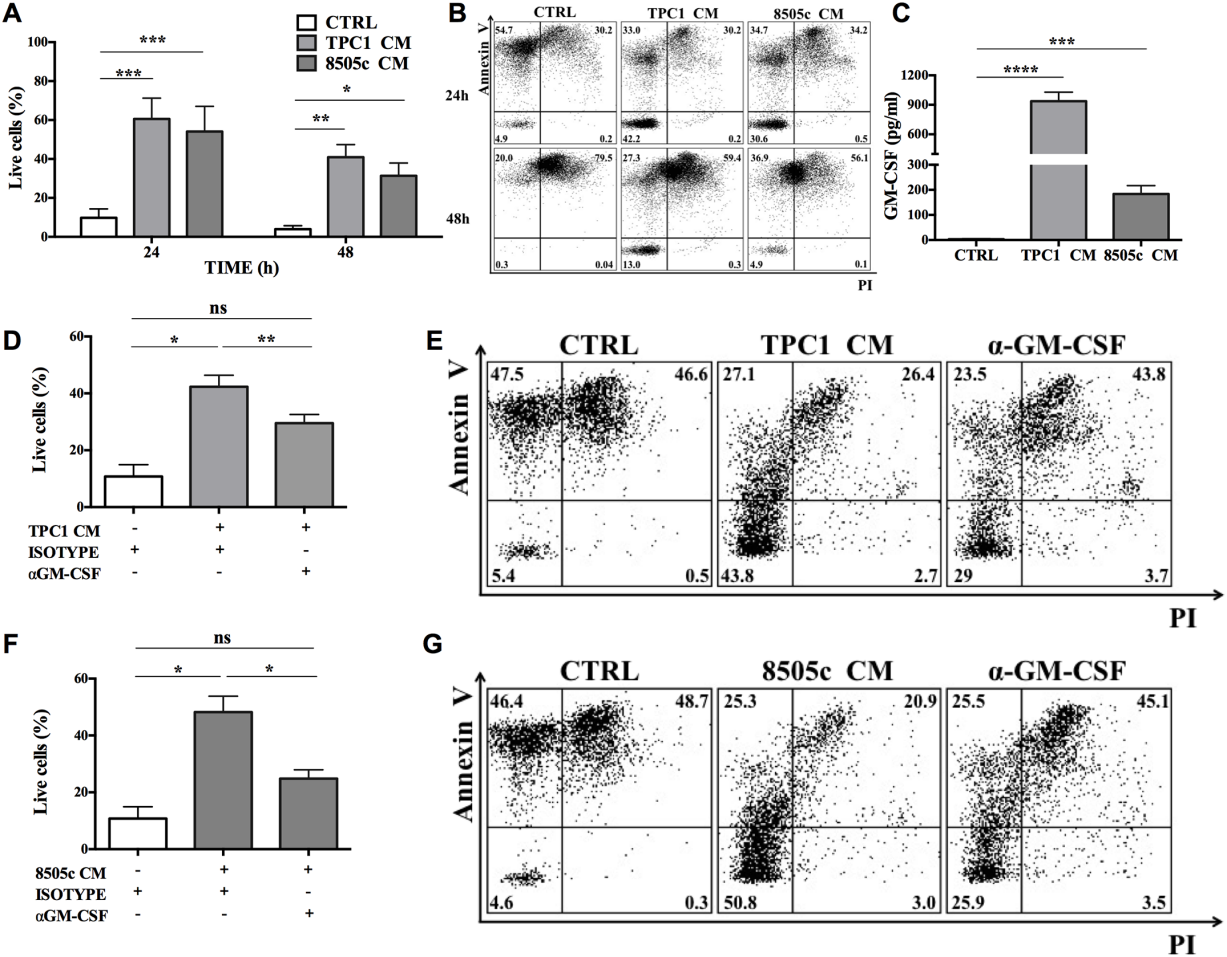
TC-derived soluble factors promoted neutrophil survival. **A.** Neutrophils were cultured in a TC-CM or the control medium. At the indicated time points, live cells were evaluated by flow cytometry with FITC-conjugated annexin V and PI. Results were expressed as percentages of live cells (mean ± SEM of five independent experiments); ***p < 0.005; **p < 0.01; *p < 0.05. **B.** Representative flow cytometric panels of dot plots of PMNs cultured in a TC-CM or control medium and stained with FITC-conjugated annexin V and propidium iodide (PI) at 24 (upper panels) and 48 (lower panels) hours. **C**. The GM-CSF release by TPC1 and 8505c cells was evaluated by an ELISA in a TC-CM or in the control medium. Results were expressed as mean ± SEM of seven independent experiments; ****p < 0.001; ***p < 0.005. **D-F.** Neutrophil survival in a TPC1-derived **(D-E)** or 8505c-derived **(F-G)** conditioned medium was evaluated in the presence of an anti-GM-CSF blocking antibody or the relative isotype control (10 μg/ml). At 24 hours, live cells were stained with FITC-conjugated annexin V and PI and analyzed by flow cytometry. **Figs E and G** illustrate representative flow cytometric panels of one out of five independent experiments. The results were expressed as mean ± SEM of five independent experiments; **p < 0.01; *p < 0.05.

We next tested whether a TC-CM could modulate PMN lifespan. To investigate the effect of TC cells on PMN survival, highly purified human neutrophils from healthy donors were cultured *in vitro* in a TC-CM or control medium. At different time points (24 and 48 hours) PMNs were stained with FITC-conjugated annexin V and propidium iodide (PI) and subjected to cytofluorimetric analysis. The presence of a TC-CM markedly increased the survival of PMNs as compared to the control medium (Fig 2A and 2B). On day 2, almost all PMNs cultured in the control medium were apoptotic (live cells represented 3.8%). In contrast, a large proportion of PMNs cultured in the presence of a TC-CM were live (40.9% ± 9%, 31.7% ± 9%; mean ± SEM) cultured in the conditioned medium from cells TPC1 and 8505c, respectively. Fig 2B illustrates representative flow cytometric panels of one out five independent experiments. Interestingly, the presence of the CM derived from the non-tumoral cell line Nthy-ori did not increase the survival of PMNs (S1 Fig). These results suggested that TC cell lines produced soluble mediators that increased PMN survival.

To dissect the molecular mechanism behind this prosurvival effect, we evaluated the presence of soluble factors known to increase the PMN lifespan in a TC-CM. GM-CSF is a well-known determinant of proliferation and differentiation of granulocytes and macrophages [49]. Of note, a large number of cell types, such as endothelial cells, T cells, macrophages, fibroblasts, mesothelial, and epithelial cells as well as tumor cells can produce GM-CSF [50]. To evaluate the molecular mechanism underlying the prosurvival effect of TC-CMs, we evaluated the presence of GM-CSF in TC-CMs by an ELISA. Cells TPC1 and 8505c constitutively produced high levels of GM-CSF, as compared to the control medium (Fig 2C). To assess the relevance of TC-derived GM-CSF in TC-CM for PMN survival, TC-CMs were depleted of GM-CSF with a neutralizing antibody. PMNs were purified and cultured in a TC-CM or the control medium in the presence of an anti-GM-CSF blocking antibody or the relative isotype control. After 24 hours, PMNs were stained with FITC-conjugated annexin V and PI and subjected to cytofluorimetric analysis. Of note, a blocking antibody, anti-GM-CSF, significantly inhibited the prosurvival effect of CM (Fig 2D and 2F). Fig 2E and 2G illustrate representative flow cytometric panels of one out of five independent experiments. Collectively, these data suggested that TC-CMs markedly improved PMN survival because of the presence of GM-CSF.

### TC-derived soluble factors induced PMN activation

**Fig 3:**
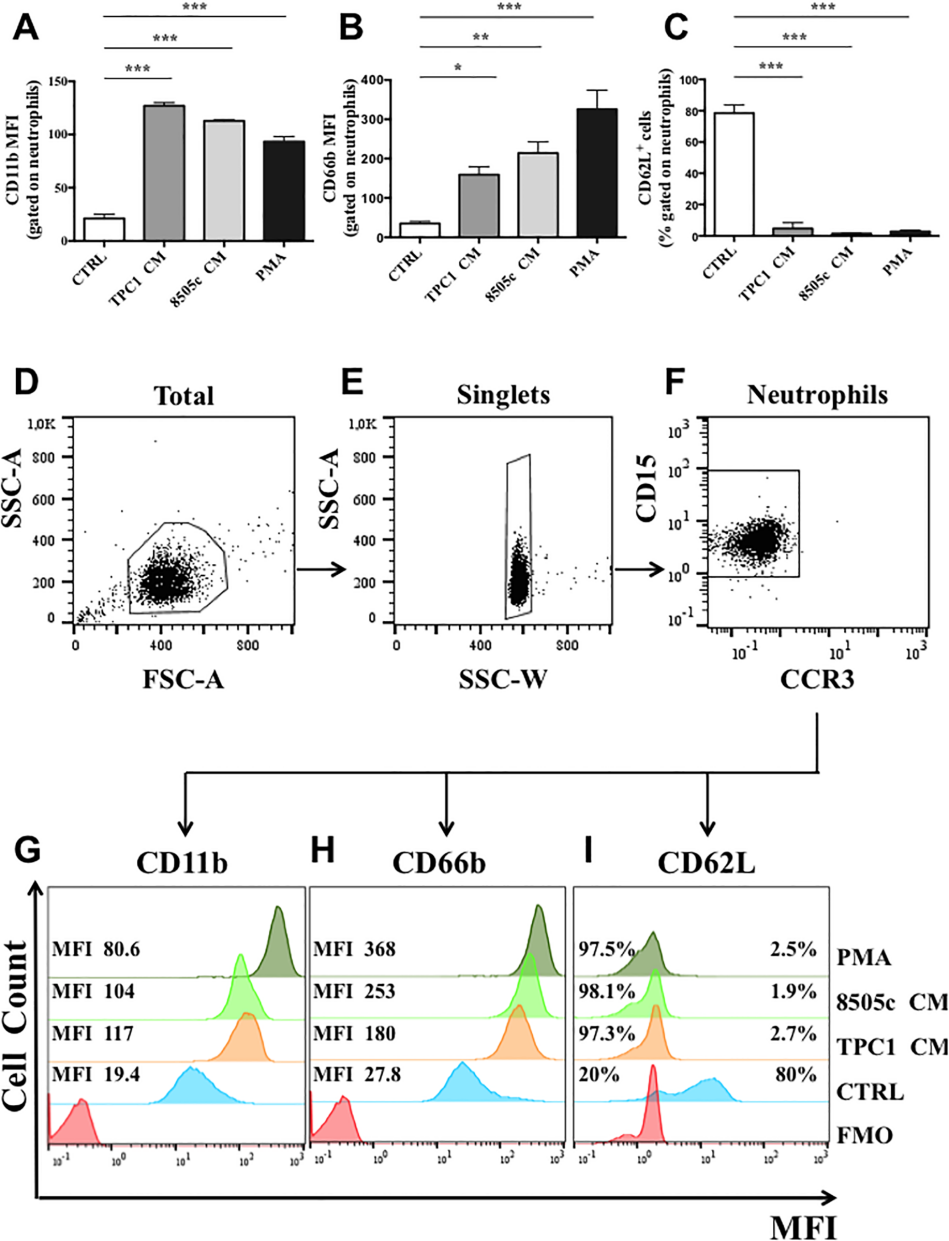
TC-derived soluble factors induced activation of neutrophils. **A–C.** Neutrophils were stimulated with a TC-CM or the control medium for 90 minutes, stained for neutrophil activation markers CD11b **(A)**, CD66b **(B)**, and CD62L **(C)** and subjected to cytofluorimetric analysis. The results were expressed as mean fluorescence intensity or percentages of positive cells gated on neutrophils (mean ± SEM of five independent experiments); ***p < 0.005, **p < 0.01, *p < 0.05. **D-F.** Representative flow cytometric panels with respect to the gating strategy of total cells **(D)**, singlets **(E)** and CD15+ CCR3- neutrophils **(F)**. **G-I.** Representative histograms illustrating mean fluorescence intensity (MFI) and cell counts for CD11b **(G)**, CD66b **(H)** and CD62L **(I)** for one out of five independent experiments. MFI = mean fluorescence intensity; FMO = fluorescence minus one.

To determine whether TC-derived soluble factors activate human PMNs, we determined CD11b, CD66b, and CD62L (L-selectin) expression on PMNs by flow cytometry [51,52]. PMNs were stimulated with PMA (as a positive control), with a TC-CM, or with the control medium. PMNs were then stained with antibodies against CD11b, CD66b, and CD62L and evaluated by flow cytometry. Under basal conditions, neutrophils showed minimal expression of CD11b and CD66b, which rapidly increased after incubation with inflammatory agonists, such as PMA (Fig 3A and 3B). TPC1 and 8505c conditioned media also induced CD66b and CD11b upregulation (Fig 3A, 3B, 3G and 3H). On the contrary, under resting conditions, PMNs highly expressed CD62L. In the presence of a TC-CM, the expression of selectin decreased, similarly to the proinflammatory control PMA (Fig 3C and 3I). Collectively, these data indicated that TC-derived soluble factors activated PMNs (CD66b and CD11b upregulation, CD62L shedding). Fig 3D–3I show representative flow cytometry panels for Fig 3A (CD11b), 3B (CD66b) and 3C (CD62L), with the specific gating strategy and related histograms (Fig 3G, 3H and 3I, respectively).

### TC-derived soluble factors induced MMP-9 release and ROS production

**Fig 4:**
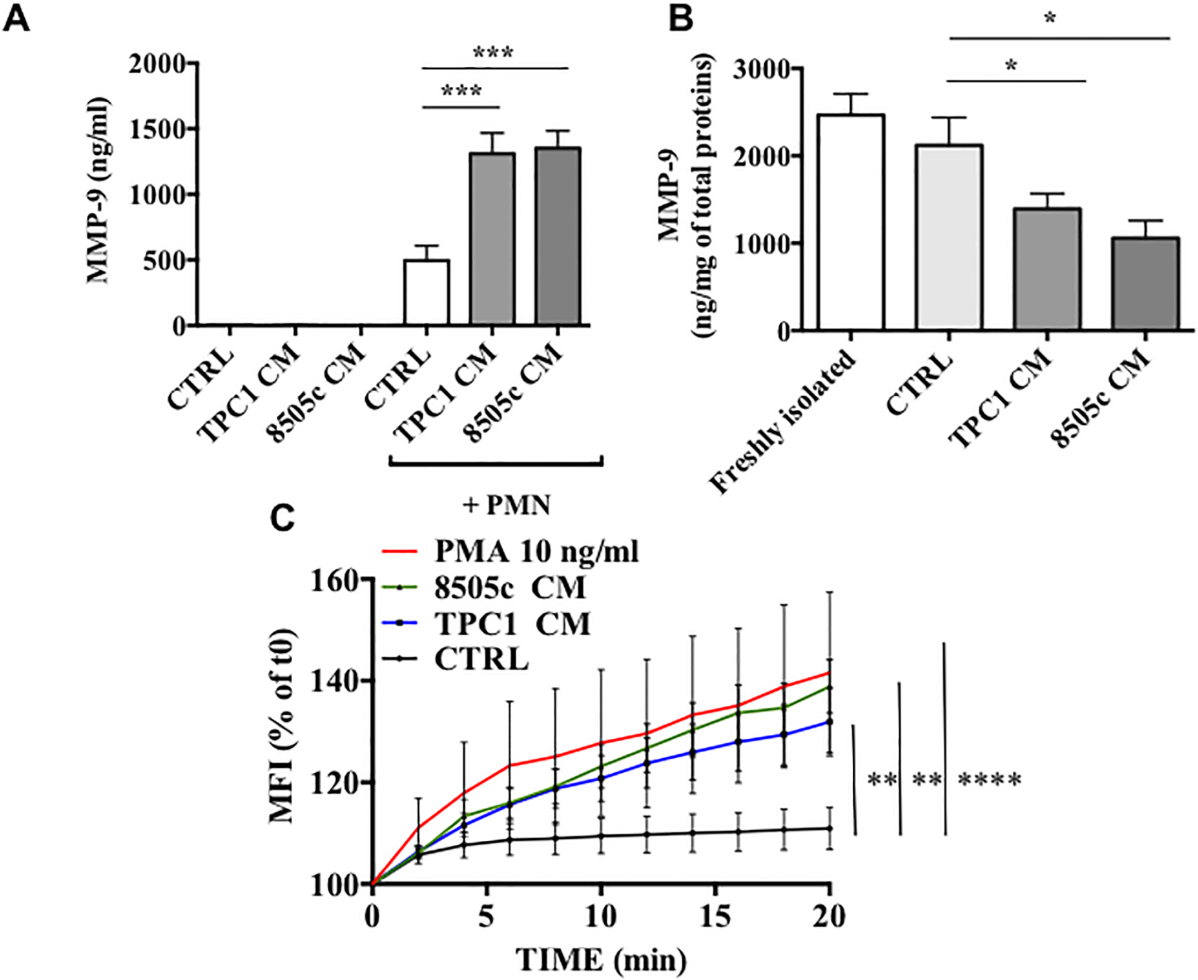
TC-derived soluble factors induced MMP-9 release and ROS production. **A and B.** Neutrophils were cultured in a TC-CM or the control medium for 18 hours. At the end of the incubation, neutrophils were harvested and centrifuged (600 × *g*, 4°C, 5 minutes), and the supernatants were collected. The extracellular release of MMP-9 from TC cell lines and neutrophils **(A)** as well as intracellular concentration of MMP-9 in neutrophils **(B)** after cell lysis (Triton X-100, 0.1%) were evaluated by an ELISA. The results were expressed as mean ± SEM of five independent experiments; ***p < 0.005; *p < 0.05. **C.** Neutrophils were incubated with 2′,7′-dichlorodihydrofluorescein diacetate (H_2_DCFDA, 10 μM, 30 minutes, 37°C), washed, and stimulated with a TC-CM or the control medium. Immediately after the stimulation, the cells were placed in a multimode microplate reader (EnSpire Multimode Plate reader, PerkinElmer), and DCF fluorescence intensity was quantitatively measured for 20 minutes at 2 minutes intervals. The results were expressed as percentages of t0 (mean ± SEM of five independent experiments); ***p < 0.005; **p < 0.01.

Besides, PMN activation was investigated by evaluation of extracellular and intracellular concentrations of MMP-9 by an ELISA. We measured the extracellular levels of MMP-9 in PMN supernatants of a TC-CM or the control medium. MMP-9 production was not detectable in TC-CMs and in the control medium (Fig 4A). MMP-9 concentration significantly increased in PMN supernatants after TC-CM stimulation (Fig 4A). In addition, PMNs cultured in a TC-CM showed reduced MMP-9 intracellular content as compared to the negative control and compared to freshly isolated cells (Fig 4B), suggesting that TC-CMs activated PMNs and mediated the MMP-9 release from PMN tertiary granules.

To test whether neutrophil activation leads to ROS production, we performed a 2′,7′-dichlorodihydrofluorescein diacetate (H_2_DCF-DA) ROS detection assay. PMNs were labeled with H_2_DCF-DA or a control medium. Fig 4C illustrates the kinetics (2 to 20 minutes) of the production of ROS induced by PMA, each TC-CM, and the control medium. ROS production by PMNs was significantly increased by TC-CMs or by PMA. By contrast, the control medium did not induce the production of ROS (Fig 4C). Collectively, these findings suggested that TC-derived soluble factors promoted the release of ROS from human PMNs.

### TC-CMs modified morphology and kinetic properties of neutrophils

**Fig 5:**
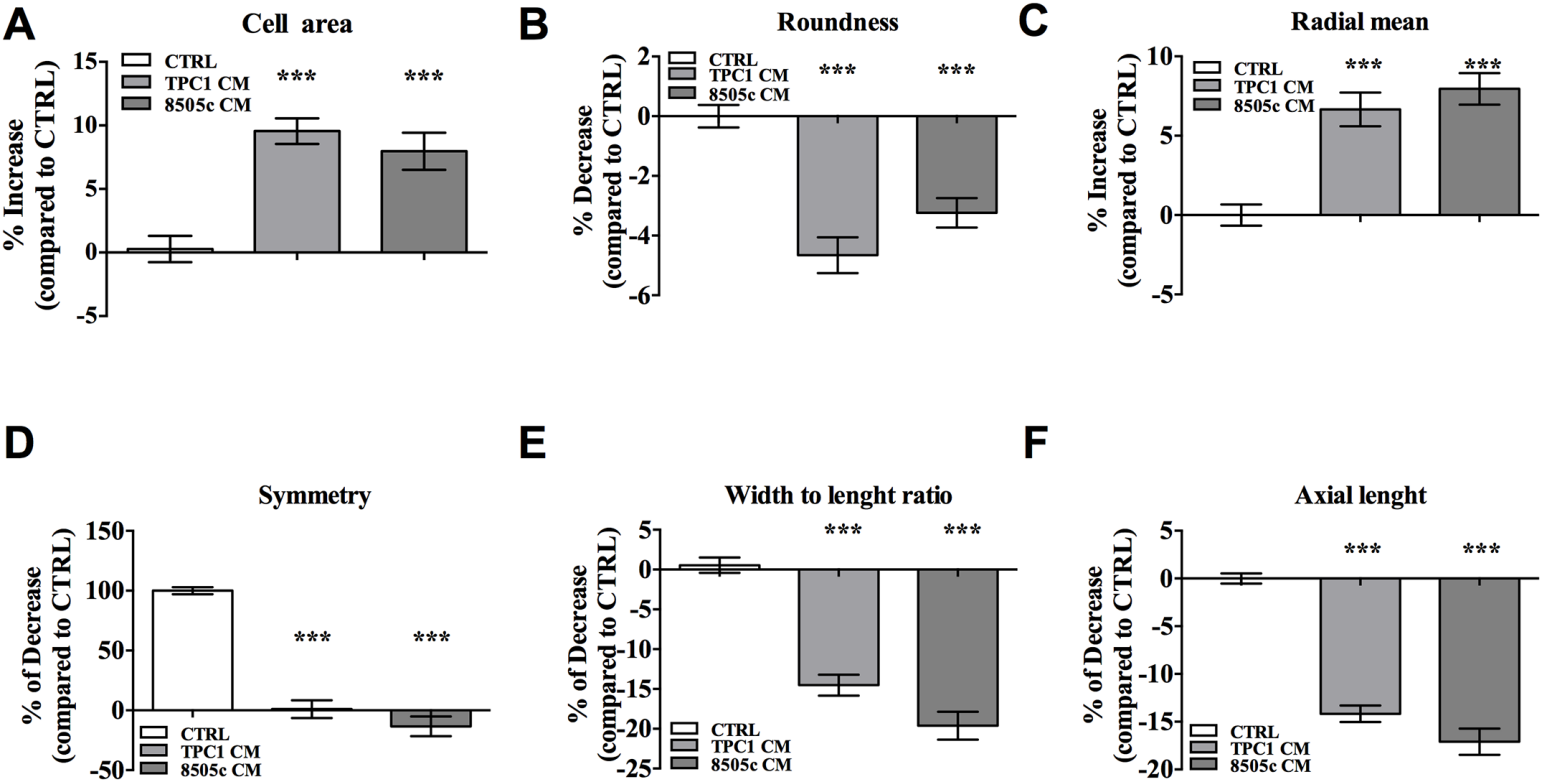
TC-CMs induced morphological changes in neutrophils. Neutrophils were stimulated with a TC-CM or the control medium for 16 hours and then were imaged by means of an Operetta high-content imaging system at 20× magnification. The images were analyzed in the Harmony software with PhenoLOGIC (PerkinElmer) and a dedicated analysis sequence (morphological properties, method STAR) to evaluate cell area **(A)**, roundness **(B)**, radial mean **(C)**, symmetry **(D)**, width-to-length ratio **(E)**, and axial length **(F)**. The results were expressed as an increase or decrease compared to the control (mean ± SEM of five independent experiments); ***p < 0.005.

Morphological cell features are related to cellular functions and have been shown to predict clinical outcomes [53]. Using the high-content imaging approach, we effectively measured and tracked changes of a number of morphological characteristics at the single-cell level and quantitatively determined these morphological feature distributions in response to the culture conditions [43,44]. To this end, PMNs were incubated with a TPC1 or 8505c TC-CM or the control medium for 16 hours at 37°C. For each individual cell, morphological characteristics were assessed. PMNs treated with a TC-CM showed increased their cell area, a greater cell radial mean, and lower width-to-length and axial length ratios and lost their roundness and symmetry (Fig 5A–5F). Thus, morphological changes in PMNs owing to cell attachment and spreading and known to occur after stimulation by inflammatory cytokines and growth factors [53], were observed when the cells were cultured in a TC-CM (Fig 5A–5F).

**Fig 6:**
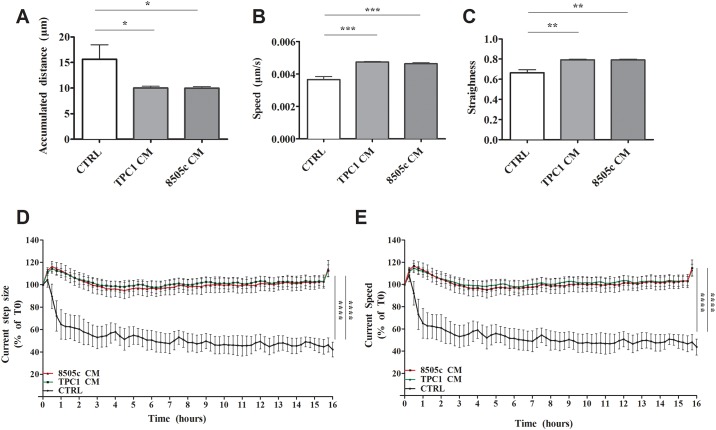
TC-derived soluble factors modified neutrophils’ kinetic properties. Neutrophils were stimulated with a TC-CM or the control medium for 18 hours. Within this time window, digital phase contrast images of 15 fields/well were captured every 15 minutes via a 20× objective in the Operetta high-content imaging system. PhenoLOGIC (PerkinElmer) was employed for image segmentation and to calculate the single-cell kinetic properties **(A)**, accumulated distance, **(B)** speed and straightness **(C)** in dedicated analysis sequence. The results were expressed as mean ± SEM of six independent experiments; ***p < 0.005; **p < 0.01; *p < 0.05. **D-E.** Timepoint analyses of time-dependent properties such as current step size (D) and current speed (E) illustrating dynamic changes of the behavior of neutrophils (in presence or absence of TC-CMs) in function of the elapsed time from treatment. The results were expressed as mean ± SEM of six independent experiments; ****p < 0.001.

In addition to evaluating morphological changes at a single time point, we detected fluctuations in cell morphology over time by time-lapse microscopy. To this end, PMNs were cultured in a TC-CM or the control medium for 16 hours at a controlled temperature (37°C) and CO_2_ levels (5%). Within this time window, digital phase contrast images were captured every 15 minutes. PMNs cultured in a TC-CM showed a reduced overall accumulated distance and an increased speed and straightness (Fig 6A–6C). Moreover, timepoint analyses performed with regard of time-dependent properties such as current step size (Fig 6D) and current speed (Fig 6E), showed that, in the presence of a TC-CM, PMNs displayed increased step size and speed compared to the control medium. Taken together, these results suggest that under the influence of TC-derived soluble mediators, PMNs modified their kinetic properties, taking up less space per time unit and losing their basal random movement.

### TC-CMs induced the expression of proinflammatory and angiogenic factors by PMNs

**Fig 7:**
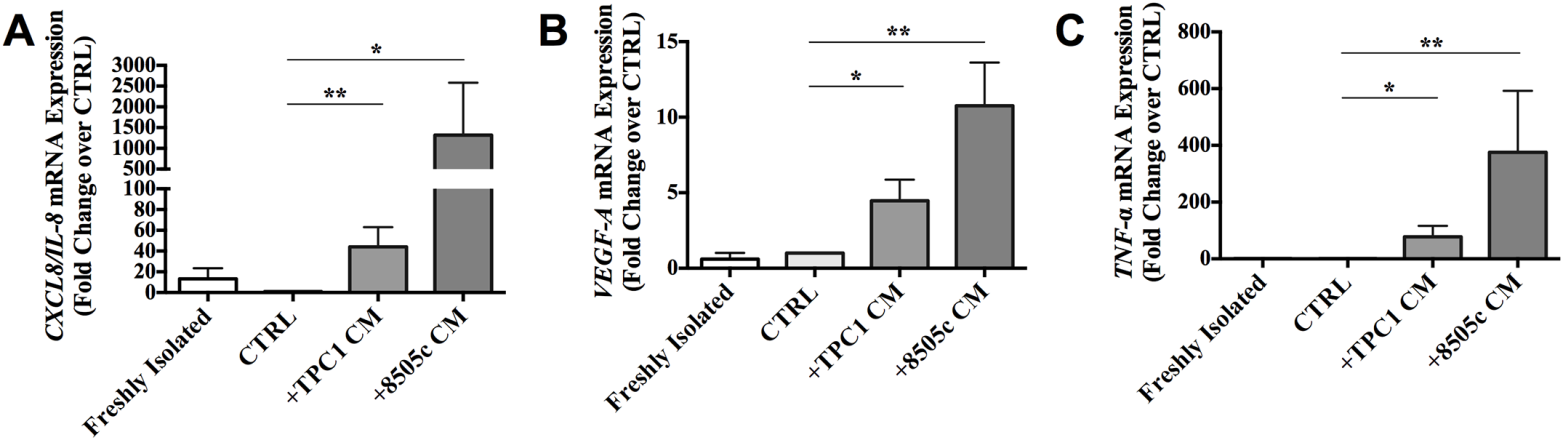
TC-derived soluble factors induced the expression of proinflammatory and angiogenic factors by neutrophils. Human neutrophils were treated with a TC-CM or the control medium for 18 hours. At the end of the incubation, the cells were harvested and lysed for RNA isolation. *CXCL8/IL-8* **(A)**, *VEGF-A* **(B)**, and *TNF-α* **(C)** mRNA levels were evaluated by real-time PCR. The results are expressed as a fold change relative to the control (mean ± SEM of six independent experiments); **p < 0.01; *p < 0.05.

We then evaluated whether the TC-CMs modified neutrophils’ gene expression. PMNs stimulated with TC-CM for 18 hours at 37°C manifested increased mRNA levels of genes encoding proinflammatory and proangiogenic molecules such as CXCL8/IL-8, vascular endothelial growth factor A (VEGF-A) and TNF-α (Fig 7A–7C). These data revealed that PMNs stimulated with TC-CM upregulated mRNA expression of various proinflammatory and angiogenic factors such as CXCL8/IL-8, VEGF-A, and TNF-α.

### Tumor-infiltrating neutrophils positively correlated with tumor size in human TC specimens

**Fig 8:**
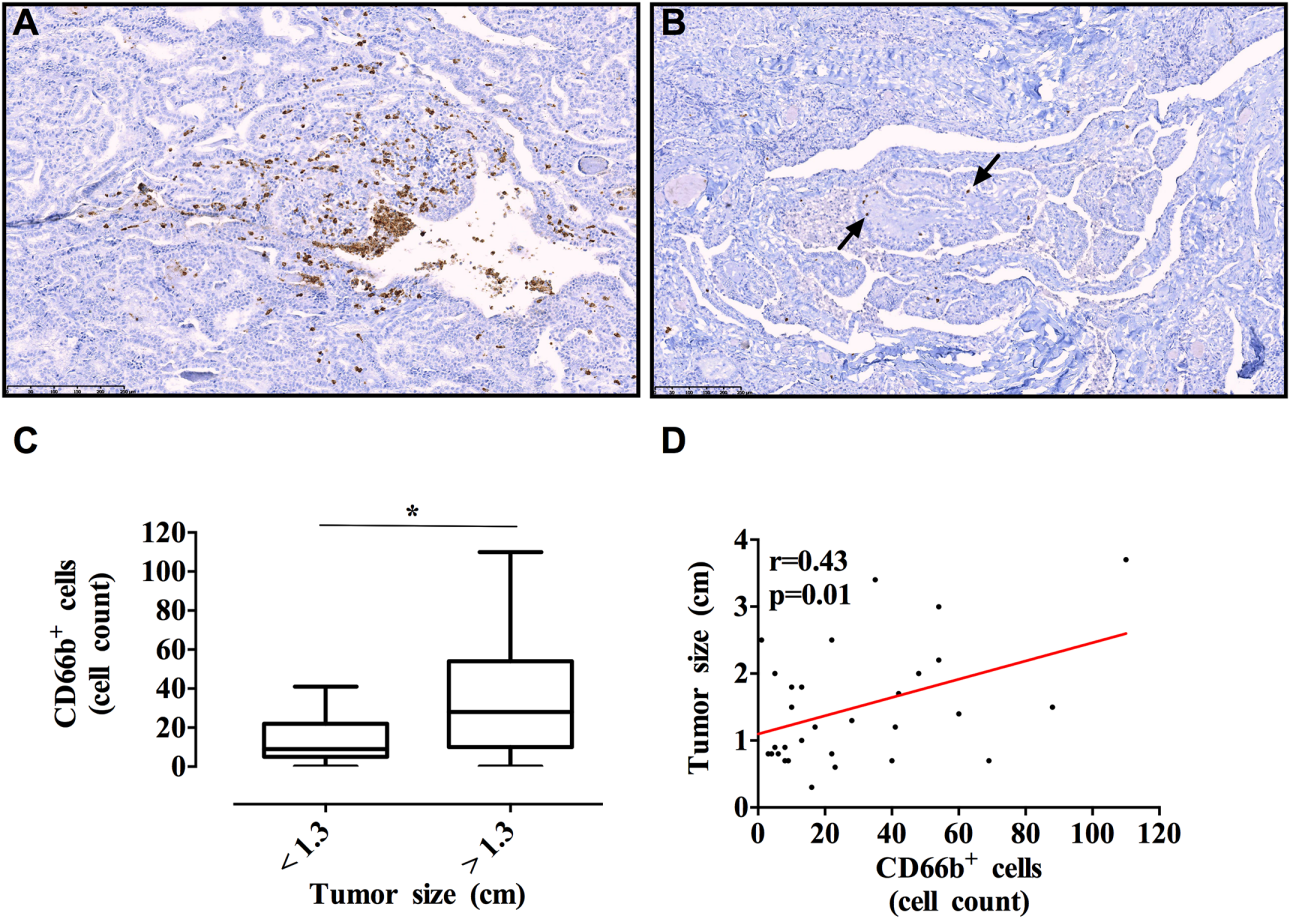
The number of tumor-infiltrating neutrophils positively correlated with tumor size in human TC specimens. **A and B.** Histological analysis of TC specimens stained with a monoclonal anti-CD66b antibody. Whole-tumor section density of CD66b^+^ neutrophils was scored at 200× magnification. Representative cases of papillary thyroid carcinomas with a high **(A)** and low **(B)** CD66b^+^ neutrophil count (arrows; hematoxylin counterstaining, 200×). **C.** CD66b^+^ neutrophil counts in tumor specimens were distributed according to the tumor size. The median value of tumor size served as a cutoff level. Results are shown as the median, the 25th and 75th percentiles (boxes), and 5th and 95th percentiles (whiskers); *p < 0.05, according to the two-tailed Mann–Whitney *U* test. **D.** Neutrophil density positively correlated with larger tumor size in TC patients (r = 0.43; p = 0.01; Pearson’s correlation test).

Our results showed that a TC-CM recruited PMNs and significantly modified their biological properties. PMNs infiltrate different types of human tumors [23]. With the exception of some case reports [54], to our knowledge, there is no information concerning the presence and significance of tumor-infiltrating PMNs in human TC. Therefore, we assessed the occurrence of CD66b^+^ PMNs by immunohistochemistry in tumorous thyroid tissue samples. To this end, a panel of 32 TC specimens was subjected to immunohistochemical analysis. The age of patients ranged from 23 to 73 years, median 41 years. Lymph node metastasis positivity was found in 46.8% (15/32) of patients during the surgical operation. An anti-CD66b monoclonal antibody, which specifically recognizes human PMNs was used [22,24,28]. Representative examples of cell staining with the anti-CD66b antibody are presented in Fig 8A and 8B. In particular, Fig 8A shows one case of mixed classic-follicular variant papillary thyroid cancer with numerous tumor-infiltrating PMNs, which are organized in clusters within the tumor tissue. Fig 8B shows one case of a classic papillary thyroid carcinoma with a few granulocytes scattered throughout the tumor tissue. An isotype-matched unrelated antibody yielded negative results (not shown). A high CD66b^+^ cell count was significantly associated with a larger tumor volume (p = 0.04, Table 1 and Fig 8C). Thus, PMN density positively correlated with larger tumor size in TC (r = 0.43, p = 0.01; Table 1 and Fig 8D). No correlations between PMN infiltration and other clinical parameters were found (Table 1).

**Table 1:** Correlation between the clinical variables and tumor-associated CD66b^+^ neutrophils in thyroid carcinoma.

	CD66b^+^ Cells (n = 32)		
	N	Median Value (IQR)	pvalue*
**Age (years)**^§^			
≥ 41	17	16 (3.5–37.5)	0.48
< 41	15	15 (8.75–55.5)	
**Sex**			
Male	9	10 (5.25–16.75)	0.22
Female	23	22 (8–48)	
**Histotype**			
Adenomas	5	13 (2.5–66)	0.59
Carcinomas	27	17 (8–41.5)	
**Mutational status**			
Wild Type	5	40 (13–60)	0.1
Mutated	27	16 (5–35)	
**Tumor size (cm)** ^§^			
≥ 1.3	17	28 (10–54)	**0.04**
< 1.3	16	9 (5–22)	
**T stage**			
T1-T2	25	19.5 (8–45)	0.1
T3-T4	7	9 (1.25–14.5)	

IQR = interquartile range;

^§^ Median value;

* Mann–Whitney *U* test

## Discussion

In this study, we investigated the possible involvement of PMNs in TC. We found that soluble factors derived from human TC cells can profoundly influence several characteristics of human PMNs. TC-CMs induced PMN chemotaxis through a release of CXCL8/IL-8, which acts on its cognate receptors CXCR1 and CXCR2 expressed on PMNs. Each TC-CM significantly increased PMN survival via a release of GM-CSF by TC cells. In addition, each TC-CM induced PMN activation (CD11b and CD66b upregulation and CD62L shedding) and profoundly modified PMN morphology and kinetic properties. Furthermore, each TC-CM induced the production of ROS, expression of proinflammatory and angiogenic factors (CXCL8/IL-8, VEGF-A, and TNF-α), and a release of MMP-9. Moreover, the density of tumor-infiltrating PMNs correlated with TC size.

A number of experimental pieces of evidence have proved that cancer-related inflammation promotes tumor initiation and progression, helping cancers to acquire all the hallmark capabilities, including evasion of immunosurveillance [55,56]. Solid tumors are characterized by an inflammatory profile and the presence of infiltrating immune cells, which together with stromal cells and blood and lymphatic vessels constitute the TME [57,58,59].

Some studies have extensively addressed the function of cells of innate immunity and adaptive immunity in TC [4] and other tumor types [23,59,60,61,62]. In particular, TAMs, dendritic cells, tumor-associated mast cells, myeloid-derived suppressor cells, natural killer (NK) cells, invariant natural killer cells, and CD4^+^ and CD8^+^ T cells have been shown to play a role in TC [4,63,64].

PMNs are canonically associated with acute inflammation where they exert a pivotal action against extracellular pathogens [19,65] and for wound repair [66]. Nonetheless, compelling evidence points to a major involvement of PMNs in different types of cancer [22,24,25,28,30,34,35,67], even though their functions are still a matter of debate, and a dual role of neutrophils in tumor biology has been described [59,68,69,70].

Several studies have examined the NLR [39,71,72]; however, these studies have limited relevance because there is increasing evidence of profound differences between peripheral-blood PMNs and TANs [34]. By contrast, to our knowledge, no studies are so far available on the occurrence, significance, and functional roles of neutrophils in human TC. Our results support the observation that human neutrophils can be activated in response to components of the TME in human TC [29,73]. To the best of our knowledge, our study is the first to reveal the presence of neutrophils in human TC, the association between neutrophil infiltration and tumor size in TC patients, and the plasticity of neutrophils under the influence of TC-derived soluble mediators. Indeed, we found that TC cells produce soluble factors able to recruit, prolong the lifespan, and to activate neutrophils. We confirmed that TC cells constitutively produce CXCL8/IL-8 and, accordingly, conditioned media from TC cells exerted chemotactic activity toward neutrophils in a CXCR1/2-dependent manner. In addition, TC cells activated PMNs and prolonged their survival via the production of GM-CSF. Moreover, we demonstrated that TC-CMs induced profound morphological and functional changes of human PMNs.

We found that a TC-CM can prolong the survival, increase activity, and ROS production of human PMNs. These findings suggest that PMNs can acquire a cytotoxic antitumor phenotype under the influence of a TC-CM [34,74]. Quite recently, however, activated neutrophils, with reduced apoptosis and increased ROS production were described in chronic leukemia and non–small cell lung cancer and were found to correlate with poor prognosis among the patients, suggesting that functional activation is not necessarily related to an antitumor phenotype [75,76].

Neutrophils are the main producers of ROS, which are the major antimicrobial tool for these cells. ROS participate in neutrophils’ cytotoxic activity against cancer cells [35,74]. Nevertheless, enhanced ROS production by neutrophils limits the NK cell–mediated antibody-dependent cytotoxicity against leukemic cells during anti-CD20 treatment, induces DNA mutations and genotoxicity, and favors drug resistance and systemic T-cell and NK-cell dysfunction [77,78,79]. Collectively, these findings suggest that neutrophil-derived ROS can exert a protumorigenic action. Further studies are needed to test whether different ROS released from activated neutrophils exert a pro- or antitumorigenic activity in TC.

PMNs activated by a TC-CM are a major source of several protumorigenic and angiogenic factors (i.e. VEGF-A, CXCL8/IL-8, and MMP-9) which are known players in cancer-related inflammation [33,80,81,82]. Indeed, neutrophil-derived MMP-9 induces the release of VEGF from the ECM, and neutrophils have been identified as the major source of MMP-9 in different types of human cancer [81,83,84]. Moreover, MMP-9 is released by neutrophils in a TIMP1-free manner, thus providing a powerful proangiogenic factor [85]. Moreover, in a murine model of transplantable melanoma and fibrosarcoma, TANs are the main regulators of angiogenesis and tumor growth because of the expression of VEGF and MMP-9 [29], suggestive of acquisition of a protumor phenotype. Nevertheless, whether a similar neutrophil polarization exists in humans still needs to be assessed.

Of note, we found a correlation between tumor-infiltrating neutrophils and human TC volume. Finally, preliminary experiments indicated that neutrophils promoted the proliferation of TC cell lines (data not shown). Taken together, our findings support the hypothesis that TANs play a protumorigenic role in human TC.

Although our results indicate for the first time possible involvement of neutrophils in human TC, further research is needed to understand their role in tumor initiation and progression. In particular, additional studies are needed to understand which mediators are responsible for TC-driven neutrophil activation as well as to elucidate the mechanisms by which these “tumor-educated neutrophils” can promote TC cell proliferation. Additional studies are needed to understand whether TAN density correlates with clinical parameters (i.e., survival) in different types of TC.

Insights into the molecular and cellular mechanisms underlying PMN infiltration in the TME in various types of TC may lead to the identification of new diagnostic and prognostic markers and perhaps novel therapeutic targets in this frequent endocrine cancer.

## Supporting information

S1 Fig TC-derived soluble factors promoted neutrophil survival. Neutrophils were cultured in a TC-CM, Nthy-ori-CM or the control medium. At the indicated time points, live cells were evaluated by flow cytometry with FITC-conjugated annexin V and PI. Results were expressed as percentages of live cells (mean ± SEM of four independent experiments); ****p < 0.001;*p < 0.05; ns = not significant.
(TIFF)
