# Supplementary figures and images for: Potential involvement of neutrophils in human thyroid cancer
Source: PLoS One. 2018 Jun 28;13(6):e0199740. doi: 10.1371/journal.pone.0199740 (PMC6023126; doi:10.1371/journal.pone.0199740)

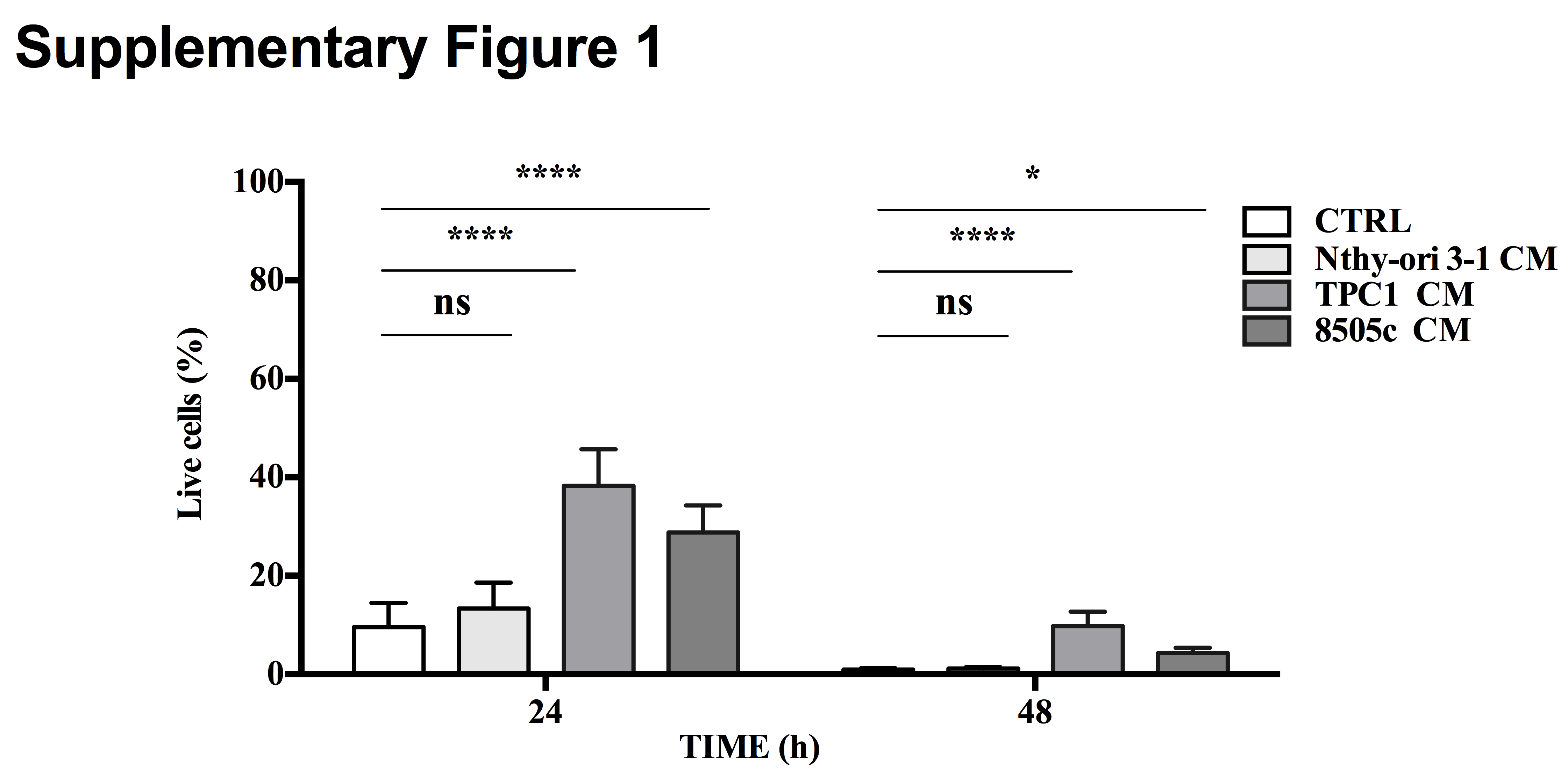

Supplement: S1 Fig — Neutrophils were cultured in a TC-CM, Nthy-ori-CM or the control medium. At the indicated time points, live cells were evaluated by flow cytometry with FITC-conjugated annexin V and PI. Results were expressed as percentages of live cells (mean ± SEM of four independent experiments); ****p < 0.001;*p < 0.05; ns = not significant. (TIFF) [file pone.0199740.s001.tiff]
